# The role and function of CTLA-4 in Sjögren’s syndrome

**DOI:** 10.3389/fimmu.2026.1868113

**Published:** 2026-07-23

**Authors:** Ji Wen, Lingshu Zhang, Linshen Xie, Dingzi Zhou, Menglin Chen

**Affiliations:** 1 Department of Rheumatology and Immunology, West China Hospital, Sichuan University, Chengdu, Sichuan, China; 2 Poisoning Department/Nephrology Department, West China Fourth Hospital Sichuan University, Chengdu, Sichuan, China; 3 Occupational Health and Occupational Medicine Key Laboratory of Sichuan Provincial Health Commission, West China School of Public Health and West China Fourth Hospital, Sichuan University, Chengdu, China

**Keywords:** abatacept, cytotoxic T lymphocyte-associated antigen-4 (CTLA-4), immune checkpoint, Sjögren’s syndrome (SS), targeted immunotherapy

## Abstract

Cytotoxic T lymphocyte-associated antigen-4 (CTLA-4) is a key immune checkpoint involved in immune regulation. CTLA-4 inhibitors have been widely used in cancer therapy and have attracted increasing attention in autoimmune diseases such as Sjögren’s syndrome (SS) in recent years. CTLA-4 modulates multiple immune processes, including genetic susceptibility, immune cell subsets, and inflammatory cytokine levels. CTLA-4, together with co-inhibitory receptors including Programmed cell death protein 1 (PD-1), T cell immunoreceptor with Ig and ITIM domains (TIGIT) and T cell immunoglobulin and mucin domain-containing protein 3 (TIM-3), forms a multi-layered immunosuppressive network. It modulates the balance of T cell subsets, regulates B cell receptor (BCR)-related pathways and downstream molecules such as Bruton’s tyrosine kinase (BTK), and thereby orchestrates cellular and humoral immunity. CTLA-4 inhibitors used in cancer immunotherapy may trigger immune-related adverse events resembling Sjögren’s syndrome. Abatacept, a CTLA-4-Ig fusion protein that blocks T-cell costimulation, has been investigated as a therapeutic candidate for primary Sjögren’s syndrome (pSS). Open-label clinical studies consistently indicate that abatacept reduces disease activity, improves salivary gland function, and alleviates systemic symptoms. However, findings from randomized controlled trials remain inconsistent: some show no significant clinical benefit compared with placebo, whereas others demonstrate improvements in clinical indices, patient-reported outcomes, and laboratory parameters. Currently, the precise mechanism of CTLA-4 in the pathogenesis of Sjögren’s syndrome\ remains incompletely clarified, and high-quality evidence supporting CTLA-4-targeted therapy is still limited. Further research is needed to validate the therapeutic value and optimal application of abatacept and other CTLA-4-modulating agents in Sjögren’s syndrome.

## 1 Introduction

Sjögren’s syndrome (SS) is a systemic autoimmune disease characterized by exocrine gland lymphocytic infiltration, causing xerostomia, xerophthalmia, and extraglandular manifestations, which severely impair quality of life. Its pathogenesis involves genetic susceptibility and immune dysregulation but remains incompletely elucidated. There is no optimal treatment for primary Sjögren’s syndrome (pSS). Evidence-based drugs with proven disease-modifying efficacy are lacking. Current therapies are mostly symptomatic and empirical, probably with heterogeneous responses, leaving an urgent unmet clinical need.

**Figure 1:**
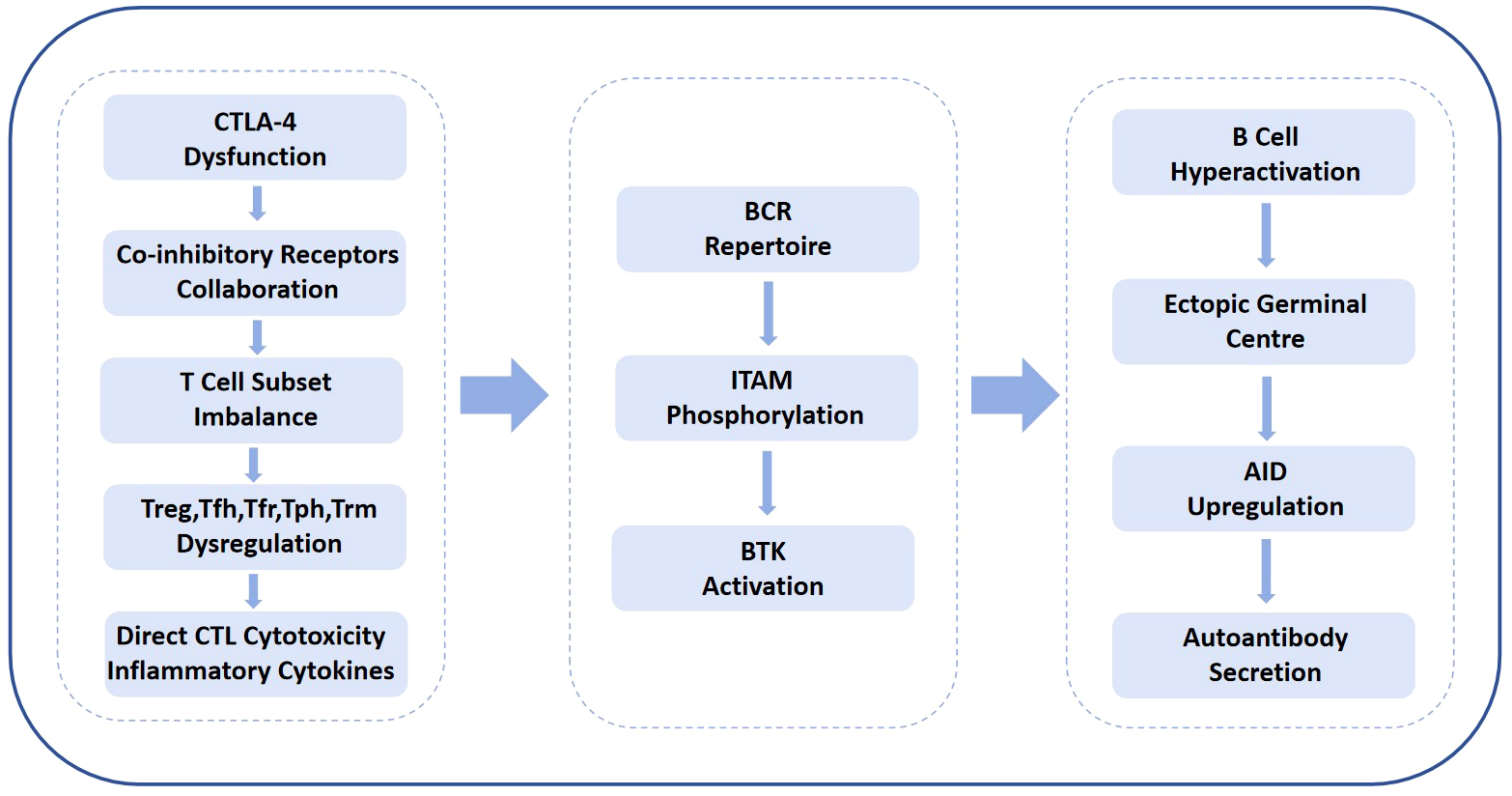
CTLA-4 dysfunction drives T and B cell dysregulation in pSS. Dysregulated CTLA-4 signaling, together with dysfunction of other co-inhibitory receptors, disrupts immunoinhibitory networks, triggering disrupted homeostasis of Treg, Tfh, Tfr, Tph and Trm cell subsets. Abnormal T cell function further leads to enhanced direct CTL cytotoxicity and release of abundant inflammatory cytokines. Downstream, altered BCR repertoires initiate ITAM phosphorylation and subsequent BTK activation, resulting in B cell hyperactivation. This cascade sequentially induces ectopic germinal center formation, elevated AID expression, and excessive autoantibody secretion. AID, activation-induced cytidine deaminase; BCR, B cell receptor; BTK, Bruton’s tyrosine kinase; CTL, cytotoxic T lymphocyte; CTLA-4, cytotoxic T-lymphocyte-associated protein 4; GC, germinal center; ITAM, immunoreceptor tyrosine-based activation motif; pSS, primary Sjögren’s syndrome; Tfh, T follicular helper cell; Tfr, T follicular regulatory cell; Tph, T peripheral helper cell; Trm, tissue-resident memory T cell; Treg, regulatory T cell.

Cytotoxic T lymphocyte-associated antigen-4 (CTLA-4) is a core inhibitory immune checkpoint that suppresses excessive T-cell activation. CTLA-4, together with co-inhibitory receptors including Programmed cell death protein 1 (PD-1), T cell immunoreceptor with Ig and ITIM domains (TIGIT) and T cell immunoglobulin and mucin domain-containing protein 3 (TIM-3), forms a multi-layered immunosuppressive network. It modulates the balance of T cell subsets, regulates B cell receptor (BCR)-related pathways and downstream molecules such as Bruton’s tyrosine kinase (BTK), and thereby orchestrates cellular and humoral immunity. Dysregulated CTLA-4 signaling modulates regulatory T(Treg)/follicular helper T cell(Tfh)/peripheral helper T(Tph)/follicular regulatory T(Tfr)/tissue-resident memory T(TRM) cell subgroups homeostasis and autoantibody production in pSS (**Figure 1**). While CTLA-4 inhibitors have revolutionized cancer immunotherapy, they can induce SS-like immune-related adverse events. Abatacept, a CTLA-4-Ig fusion protein, shows consistent efficacy in open-label studies but yields conflicting results in randomized controlled trials.

This review summarizes current advances in CTLA-4’s role in SS pathogenesis, evaluates abatacept’s preclinical and clinical evidence, analyzes potential reasons for trial discrepancies, and highlights key unresolved questions, aiming to inform the development of optimized targeted therapies for SS.

## 2 Main text

### 2.1 Molecular structure and function of CTLA-4

Effector T-cells achieve full activation and complete terminal differentiation via a dual-signal regulatory mechanism. Within the co-stimulation network, CD28 and CTLA-4 jointly regulate T-cell function yet exert opposing biological effects. CD28 delivers stimulatory signals to trigger T-cell activation, whereas CTLA-4 functions as a negative regulator to limit excessive immune responses.

Also known as CD152, CTLA-4 belongs to the immunoglobulin superfamily and is a well-documented leukocyte differentiation antigen. It binds B7.1 (CD80) and B7.2 (CD86) with far higher affinity than CD28. On antigen-presenting cells, CTLA-4 competes for ligand binding sites. This competition disrupts CD28-mediated co-stimulation and activates intracellular inhibitory cascades, slowing the proliferation of T-cells (1). As a classic immune checkpoint, CTLA-4 has become a major research focus in tumor immunology. All therapeutic agents developed to block CTLA-4 activity are classified as immune checkpoint inhibitors (ICIs).

Accumulating research demonstrates that dysregulated CTLA-4 signaling participates in the onset and progression of SS and other autoimmune disorders (2). SS is a chronic autoimmune disease characterized by persistent lymphocytic infiltration and inflammation of exocrine glands. Its pathogenesis arises from multiple factors, including genetic predisposition, imbalanced immune cell function and abnormal cytokine production. Altered CTLA-4 signaling is confirmed to be closely associated with this pathological process (3, 4). Clinical observations show that peripheral T-cells from patients with pSS express reduced CD28, alongside a marked expansion of CTLA-4-positive cell populations (5).

Salivary gland epithelial cells (SGECs) are the primary target cells damaged in SS lesions. Kapsogeorgou and colleagues reported that SGECs derived from SS patients express B7 family proteins. On these cells, B7.2 retains the capacity to bind CD28 and initiate co-stimulation, while its binding affinity for CTLA-4 is significantly diminished (6). T-cells participate in pathological processes throughout the entire course of SS. At the early disease stage, abundant lymphocytes infiltrate gland tissues and form a long-standing inflammatory microenvironment, which eventually causes irreversible damage to exocrine glands and impairs secretory function (7).

Serological examinations have detected natural autoantibodies targeting CTLA-4 in 13.3% of pSS patients. Comparatively, autoantibodies against CD28, B7.1 and B7.2 are rarely detected in this patient cohort (8). These findings indicate that SS pathogenesis involves not only CTLA-4-associated T-cell dysfunction, but also B-cell-driven humoral immune disorders. These two pathological mechanisms are elaborated in the following sections.

### 2.2 Distinct therapeutic strategies targeting CTLA-4 for tumors and autoimmune diseases

The CTLA-4/CD28 signaling axis is widely investigated in both oncology and autoimmune disease research. Monoclonal antibodies against CTLA-4 interfere with the interaction between this receptor and B7 ligands. This intervention reverses immune suppression and restores anti-tumor immune activity. For autoimmune diseases, treatment strategies follow a different route: enhancing CTLA-4-mediated immune inhibition can suppress aberrantly activated immune reactions. Researchers must design targeted intervention regimens for different diseases when modulating the CTLA-4 checkpoint pathway.

CTLA-4 modulators are mainly divided into neutralizing antibodies and recombinant fusion proteins, both of which have undergone comprehensive preclinical and clinical evaluation. Ipilimumab is the first anti-CTLA-4 monoclonal antibody approved by the US Food and Drug Administration (FDA), with its initial indication for melanoma (9). Subsequent clinical trials validated its reliable efficacy against a broad range of solid malignancies. Combining anti-CTLA-4 agents with other ICIs such as anti-PD-1 antibodies can further boost anti-tumor effects and induce tumor regression (2).

Different from CTLA-4 blocking antibodies, abatacept is a recombinant fusion protein designed to enhance endogenous immune suppression. It obtained official approval for rheumatoid arthritis (RA) earlier than ipilimumab. This engineered protein consists of the extracellular domain of CTLA-4 fused to the Fc fragment of human IgG1. It selectively binds B7 ligands to suppress pathological immune activation. A large body of clinical data has confirmed its efficacy across multiple immune-mediated rheumatic diseases (IMRDs), including psoriatic arthritis, juvenile idiopathic arthritis and inflammatory myopathy (10), giant cell arteritis (11), and anti-neutrophil cytoplasmic antibody (ANCA)-associated vasculitis (12). Although abatacept has been applied to various rheumatic conditions (13), further clinical evidence is still required to confirm its therapeutic value in pSS.

In cancer therapy, CTLA-4 inhibitors break immune tolerance and reinforce anti-tumor immune defense. Nevertheless, excessive immune activation triggered by these drugs may induce new autoimmune complications such as SS. Immune-related adverse events (irAEs) linked to ICIs have drawn widespread clinical attention. Among various ICIs, CTLA-4 agents are more likely to induce irAEs (14). Rheumatic complications remain relatively uncommon within the spectrum of irAEs, mainly manifesting as inflammatory arthritis, rheumatic myalgia, giant cell arteritis, inflammatory myopathy and secondary Sjögren’s syndrome (sSS) (15).

Blocking CTLA-4 eliminates its intrinsic immunosuppressive function, which explains the high incidence of irAEs during anti-CTLA-4 treatment. The emergence of sSS in cancer patients receiving CTLA-4 inhibitors provides strong clinical evidence: impaired CTLA-4/CD28 signaling serves as a key driver for pSS development.

### 2.3 CTLA-4 inhibitors and immune-related adverse events resembling SS

CTLA-4 antibodies were developed to treat various malignancies, including melanoma, lung cancer, mesothelioma and other solid tumors. These agents deliver robust anti-tumor activity, yet they frequently cause irAEs. Such toxicities affect 60-65% of treated patients (16). Germline mutations that disrupt CTLA-4 signaling also lead to a broad spectrum of autoimmune manifestations: cytopenias, arthritis, intestinal inflammation and sSS are all commonly observed.

ICIs other than CTLA-4 antibodies can also trigger autoimmune complications during cancer treatment, with SS being one frequent presentation (17, 18). Clinical data reveal that sSS develops in 9.4% of patients receiving combined anti-CTLA-4 and anti-PD-1 therapy; the incidence drops to merely 1.4% among those receiving CTLA-4 monotherapy (18). PD-1/Programmed death ligand 1 (PD-L1) blockade acts as the main driver behind ICI-associated sialadenitis and SS, while CTLA-4 inhibition only plays a supportive, synergistic role. Multiple clinical observations support this conclusion. According to Ramos-Casals et al. (19), no cases of ICI-related SS were linked to CTLA-4 monotherapy, whereas all reported cases occurred in patients treated with PD-1/PD-L1 inhibitors. A subsequent systematic review analyzed 93 patients diagnosed with ICI-associated SS or sialadenitis; most participants had received PD-1 inhibitors for melanoma or lung cancer (20).

ICI-induced sSS differs markedly from pSS in both clinical profiles and pathological features. This secondary disorder occurs more often in elderly male patients, who typically present seronegative results alongside obvious organ-specific autoimmune lesions (19). Serum autoantibody levels are generally lower in this patient group. Pathological examination of salivary gland lesions shows sparse CD20+ B-cell infiltration, and T cells predominate within local inflammatory infiltrates (18). Within gland tissues, CD3+ T cells accumulate extensively; CD4+ cells slightly outnumber CD8+ populations, and CD20+ B cells are rarely found. In contrast, pSS is characterized by massive B-cell infiltration, frequent germinal center (GC) formation and mixed T-cell infiltration in salivary glands (21). Given the clear link between sicca symptoms, SS and blockade of the CTLA-4/PD-1 pathway, further investigations remain necessary (19).

Compared with PD-1/PD-L1 inhibitors, CTLA-4 agents carry a higher risk of grade 3–5 severe immune toxicity (22). irAEs are undesirable treatment complications, and their occurrence is often correlated with improved anti-tumor responses in certain tumor types. This association does not hold across all ICI classes. Among melanoma patients, irAE occurrence shows no correlation with the efficacy of CTLA-4 blockade. For PD-1 and PD-L1 inhibitors, however, immune adverse events consistently predict favorable clinical outcomes across multiple solid tumor types (23).

Identifying high-risk groups, preventing irAEs and reducing ICI-related immune toxicity have become key priorities for clinicians. Multiple risk factors and biomarkers can predict irAEs, with distinct predictive performance among different ICI regimens (24). Demographic and lifestyle factors raise disease susceptibility: age under 60 years, high body weight and long-term smoking are all relevant contributors. Pre-existing chronic disorders, autoimmune diseases and concurrent medications also increase overall risk. Peripheral blood parameters, cytokines, autoantibodies, HLA genotypes and non-coding RNAs serve as major predictive biomarkers.

Combined anti-CTLA-4 and anti-PD-1 treatment reduces circulating B cells. It also elevates counts of CD21lo PD-1+ memory B cells and plasmablasts. These cellular changes can effectively predict subsequent irAEs (25). CD21lo B cells express low levels of chemokine receptors required for lymphoid tissue homing. This functional trait allows them to migrate into non-lymphoid tissues and drive local inflammation. Comprehensive risk assessment and early intervention are therefore essential before initiating ICI therapy.

Glucocorticoids remain the first-line therapy for irAEs. Debate still surrounds the impact of high-dose glucocorticoids on ICI anti-tumor activity: some studies report reduced treatment potency, while others find no tangible negative effects. To date, standard guidelines for ICI-induced sSS are still lacking. Current clinical management follows protocols established for pSS, combining glucocorticoids with conventional immunosuppressive drugs.

Researchers have explored cytokine-targeted approaches to alleviate irAEs. Inhibitors against granulocyte-macrophage colony-stimulating factor (GM-CSF), interleukin-6 (IL-6), interleukin-23 (IL-23) and tumor necrosis factor (TNF) are the most widely investigated candidates. Though most relevant studies do not focus specifically on ICI-related sSS, their findings deliver valuable clinical references. In melanoma patients, ipilimumab combined with GM-CSF lowers immune toxicity and prolongs overall survival, with no changes observed in tumor response rates (26). Mouse studies demonstrated that IL-6 blockade enhances the anti-tumor effects of CTLA-4 inhibitors. Serum IL-23 levels rise after dual ICI treatment, and these concentrations correlate with irAE severity (27). Anti-IL-23 antibodies can relieve irAEs while preserving anti-tumor activity, as validated in animal models (27). Prophylactic TNF blockade also mitigates ICI-related colitis and hepatitis, and improves overall therapeutic efficacy (28).

Most ongoing research aims to block excessive inflammatory signaling, to separate ICI anti-tumor benefits from immune toxicities. Unfortunately, the majority of these strategies are still confined to preclinical work or early-phase clinical trials. Large-scale clinical trials focusing on ICI-induced sSS are urgently required to fill this research gap.

### 2.4 Distinct role and relationship between CTLA-4 and other co-inhibitory receptors in pSS

CTLA-4 and PD-1 are the most well-characterized immune checkpoint receptors; they also act as key targets for modern cancer immunotherapy. Alongside TIGIT, TIM-3 and lymphocyte-activation gene 3 (LAG-3), these molecules form a complex network that maintains immune homeostasis in humans (29). Each checkpoint functions at distinct stages of immune activation. CTLA-4 modulates early T cell activation within lymph nodes; PD-1 suppresses effector T cell activity in tumor microenvironments; TIGIT, TIM-3 and LAG-3 act as secondary immune suppressors. Dual CTLA-4 and PD-1 blockade produces potent anti-tumor effects, yet it greatly increases irAEs risk — including the onset of sSS.

ICI treatment disrupts immune tolerance and triggers sSS. Clarifying how these checkpoint molecules function during the development of pSS is essential. This section compares the biological roles of CTLA-4 and PD-1 in pSS, and briefly reviews TIGIT, TIM-3 and LAG-3, whose pathogenic roles in pSS have not been fully elucidated.

CTLA-4 and PD-1 both suppress overactive immune responses, but they work at different phases of immune reactions (30). CTLA-4 limits early T cell proliferation in lymph nodes; PD-1 restrains T cell activity in peripheral tissues. Data from tumor models showed that CTLA-4 inhibitor treatment expands effector CD4+ T cells, whereas PD-1 blockade reactivates exhausted CD8+ T cells (31).

Combined anti-CTLA-4 and anti-PD-1 therapy expands autoreactive mature naive lymphocytes in peripheral tissues. CTLA-4 mainly restricts the accumulation of these self-reactive cells, while PD-1 has minimal influence here. No available evidence suggests that either drug interferes with central B cell tolerance. Combination treatment also impairs Treg function. Tregs normally limit the expansion of autoreactive B cells, so such functional deficits partially explain why CTLA-4 and PD-1 inhibitors differ in their capacity to induce irAEs (32).

In the context of pSS pathogenesis, CTLA-4 and PD-1 target different T cell subsets. PD-1 primarily acts on Tph cells and Tfh cells. CTLA-4 modulates Tregs, Tfh cells and T resident helper (Trh) cells. Both molecules regulate B cell activity indirectly through T cell-dependent mechanisms. CTLA-4 reshapes the BCR repertoire and alters peripheral B cell tolerance. Abnormal BCR selection drives autoreactive B cell activation, a key pathological hallmark of pSS. It also modulates downstream BCR signaling pathways, thus participating in disease progression.

PD-1 is highly expressed on T lymphocytes. Its ligand PD-L1 can be detected on epithelial cells within inflamed salivary glands from pSS patients. Dysfunction of the PD-1/PD-L1 axis breaks immune tolerance and promotes SS progression (33). A reduced proportion of PD-1+ T cells has also been observed in affected patients (5). Among PD-1-expressing T cells, Tfh and Tph subsets are central to disease development.

Tfh and Tph cells represent major PD-1hi T cell populations. They share several features while carrying out distinct biological roles (34). As essential B cell helper cells, PD-1hiC-X-C motif chemokine receptor 5 (CXCR5)+ CD4+ Tfh cells and PD-1hiCXCR5− CD4+ Tph cells secrete IL-21, C-X-C motif chemokine ligand 13 (CXCL13) and inducible T-cell co-stimulator (ICOS) to support B cell activation and differentiation (35). The two subsets also differ in anatomical location: Tfh cells interact with B cells inside lymphoid organs, and Tph cells provide helper signals in inflamed peripheral tissues (36).

Ectopic germinal centers (GCs) and FcRL4+ B cells form a unique immune microenvironment in salivary gland lesions of SS patients. Tfh/Tph cells, CXCL13 and the B-cell-activating factor (BAFF)/a proliferation-inducing ligand (APRIL)-NF-κB pathway work together to induce plasmablast formation (37). Overactivated Tfh and Tph cells drive excessive B cell activity, which is a central pathological change in pSS (38). Clinical findings demonstrate that PD-1+CXCR5− CD4+ Tph cells and the Tph/Treg ratio are significantly higher in pSS patients than in healthy controls. These two biomarkers correlate closely with disease activity, serum IgG, inflammatory factors and autoantibody levels (39, 40).

PD-1 exerts diverse effects on Tfh and Tph cells. It blocks CXCR5-dependent T cell migration into lymphoid follicles, and retains Tfh cells within germinal centers to facilitate antibody affinity maturation (41). The exact role of PD-1 in Tph cells still lacks clear evidence. Even so, therapies targeting Tfh and Tph cells can relieve SS symptoms. In animal models, CD8+ T cell depletion leads to abnormal expansion of Tfh and Tph cells, accompanied by typical SS-like manifestations (42). Clinically, the Janus kinase (JAK) inhibitor tofacitinib suppresses Tfh and Tph activity and improves clinical outcomes for pSS patients (43).

Beyond CD4+ T cell subsets, PD-1 also regulates CD8+ T cells — especially tissue-resident memory T (TRM) cells, critical mediators of pSS. In patient salivary glands, CD8+ TRM cells outnumber CD4+ T cells and accumulate around glandular epithelial structures (44). Clonal expansion of T and B cells, particularly CD8+ T cells, is widely seen in pSS.

Peripheral granzyme k (GZMK)+C-X-C motif chemokine receptor 6 (CXCR6)+ CD8+ T cells act as precursors of gland-resident TRM cells. Clonal overlap between these two cell populations rises markedly in pSS (45). GZMK+ TRM cells possess strong cytotoxicity; they damage epithelial cell mitochondria and activate type I interferon signaling. This pathway links glandular injury to persistent local inflammation (46). Continuous attack from these cytotoxic cells sustains chronic inflammation in salivary glands, forming one key mechanism of pSS.

TRM cells are defined by surface markers CD103, CD49a and CD69, and do not circulate in peripheral blood. They secrete large amounts of granzyme B, interferon-γ (IFN-γ) and TNF-α, and co-express PD-1, CTLA-4, TIM-3 and other immune checkpoints (47). Studies using tumor models showed that CD103+ CD8+ TRM cells respond mainly to PD-1 blockade, while CD49a+ CD4+ TRM cells serve as major effectors for CTLA-4-targeted therapy (48).

In mouse submandibular glands, NK cells maintain local immune homeostasis via the PD-1/PD-L1 axis by restraining GZMK+ CD8+ T cell activation. This cell subset shares phenotypic features with pathogenic TRM cells in human SS. Loss of PD-1 function triggers massive clonal expansion and hyperactivation of GZMK+ CD8+ T cells. These cells upregulate LAG-3, TIGIT and CTLA-4 to limit overactive T cell receptor (TCR) signaling, and eventually develop a partially exhausted yet pro-inflammatory phenotype (49). NK cells are key regulators of glandular CD8+ T cell activity, and their functions provide new ideas for preventing and treating ICI-related irAEs.

TIGIT, TIM-3 and LAG-3 are recognized as second-generation immune checkpoints. They modulate TCR signaling, myeloid cell and B cell function, and balance T helper cell responses (29). CTLA-4 and PD-1 belong to primary checkpoint molecules, which maintain systemic immune tolerance (**Figure 1**). TIGIT, TIM-3 and LAG-3 work mainly at local inflammatory sites and act as secondary regulators (50).

Knockout mouse models further confirm their functional differences. Germline deletion of CTLA-4 or PD-1 causes severe spontaneous autoimmunity in mice. PD-1 knockout animals develop autoimmune disorders later and less frequently than CTLA-4-deficient littermates. Single knockout of TIGIT, TIM-3 or LAG-3 cannot induce autoimmune disease; their inhibitory functions only take effect under disease-susceptible genetic backgrounds (50). To date, the pathological roles of these novel checkpoints in pSS remain unclear.

TIGIT is broadly expressed on NK cells, Tregs, Tfh cells and exhausted CD8+ T cells. Its overall expression increases in pSS patients: TIGIT levels go up on T cells but drop on CD14+ monocytes (51). Reduced TIGIT expression on monocytes correlates strongly with disease activity. This change is commonly found in patients with arthralgia, fatigue, oral lesions, interstitial lung disease, positive anti-Ro52 antibodies or elevated IgG (52).

Dysregulated TIGIT expression drives excessive CD4+ T cell activation and immune imbalance in pSS (53). γδ T cells from pSS patients show higher co-expression of TIGIT and PD-1, alongside reduced cell counts. Such phenotypic changes correlate closely with clinical symptoms and serological indicators (54). Active pSS patients also have a higher percentage of CD4+ and CD8+ T cells co-expressing CD226 and TIGIT (53). In short, abnormal TIGIT expression across multiple immune cell subsets is closely linked to pSS activity and clinical manifestations.

Research on TIM-3 and LAG-3 lags far behind studies on TIGIT. T cells isolated from SS patients and NOD mice show downregulated TIM-3 expression, which contributes to disease progression (55). Clinically, the frequency of CD8+ TIM-3+ T cells correlates with serum anti-Sjögren’s syndrome antigen A (SSA) antibody levels (56).

Low deltex E3 ubiquitin ligase 1 (Deltex1) expression is also detected in T cells from SS patients. This molecular alteration is associated with multiple extra-glandular complications, including immune thrombocytopenia, vasculitis and autoimmune thyroid disease. Patients with low Deltex1 levels show higher checkpoint expression on Tregs, along with expanded inflammatory T cell populations (57). Collectively, TIM-3 and LAG-3 participate in pSS progression, but relevant research is still in the early stage. Further mechanistic and clinical work is required to clarify their immunomodulatory functions.

### 2.5 Regulation of B cells by CTLA-4

As illustrated in **Figure 1**, CTLA-4 dysfunction triggers multi-layered immune disorder involving T and B cell compartments in pSS (**Figure 1**). Dysregulated T and B cell activity underpins the pathogenesis of pSS. Clinically, this autoimmune disorder is characterized by three hallmark manifestations: robust lymphocytic infiltration within glandular tissues, elevated circulating autoantibodies and persistent hypergammaglobulinemia. Binding of BCRs to target antigens triggers phosphorylation of intracellular immunoreceptor tyrosine-based activation motifs (ITAMs). This intracellular cascade subsequently drives B cell proliferation, aberrant cellular activation and excessive autoantibody secretion.

BCR signaling dynamics vary substantially across tissues and disease stages in patients with pSS. As (58) demonstrated, combined defects in BCR repertoires, antigen binding capacity and downstream signaling pathways sustain chronic B cell hyperactivity. Cumulative genetic evidence further validates aberrant BCR signaling as a central pathogenic driver of pSS.

BCR repertoire profiles share partial similarities between pSS patients and healthy individuals. Distinct differences emerge, however, when comparing circulating B cells with lymphocytes infiltrating labial glands. Clonal expansion patterns, immunoglobulin isotypes and BCR clustering all diverge markedly between these two cellular compartments. Although the diversity of heavy and light chain complementarity-determining region 3 (CDR3) regions remains comparable across groups, the BCR κ/λ ratio in labial and parotid glands correlates positively with pSS clinical activity (59).

Plasma cell clonal expansion shows limited heterogeneity in pSS. In contrast, salivary gland-resident memory B cells exhibit contracted BCR diversity, a phenotypic hallmark of *in situ* clonal proliferation. Shared B cell clones have been identified across multiple peripheral and glandular B cell subsets. Dominant clonotypes predominantly target 60-kDa Sjögren’s syndrome antigen A (Ro60), 52-kDa Sjögren’s syndrome antigen A (Ro52) and centromere antigens; a large panel of unclassified autoantibodies remains to be characterized (60).

Transcriptomic profiling reveals widespread activation of multiple signaling cascades in pSS. Pathways governing antigen processing, antigen presentation, BCR and TCR signaling are all markedly upregulated (61). Single-cell BCR repertoire analysis indicated no significant disparities in clonal expansion or CDR3 characteristics between patients and healthy controls (61).

The type I interferon axis is well-established in pSS research. Beyond this classical pathway, aberrant B cell function also contributes to disease progression via remodeled BCR clonotypes and rearranged immunoglobulin gene segments (40). B cell transcriptomes undergo dynamic shifts throughout disease progression. Genes associated with cellular stress and early activation predominate at initial stages, while genes mediating immunoglobulin synthesis and plasma cell differentiation become dominant as disease advances.

Patients diagnosed with pSS carry an elevated risk of developing non-Hodgkin lymphoma (NHL). Persistent BCR activation is a shared feature between pSS and diffuse large B cell lymphoma (DLBCL), as documented by Mohammadnezhad’s study (62). This conserved pathway highlights the biological importance of BCR signaling, and warrants further exploration into the modulatory effects of CTLA-4 on this cascade.

Pharmacological blockade of CTLA-4 remodels overall BCR repertoires and expands autoreactive B cell clones, a phenotype closely linked to human autoimmunity. These autoreactive lymphocytes recognize antigens derived from endocrine and peripheral tissues, and also cross-react with tumor-associated antigens. Such dual antigen reactivity confers potent anti-tumor immunity but simultaneously induces irAEs (32). To date, the molecular mechanisms by which abatacept modulates BCR signaling have not been fully elucidated, and may differ from conventional CTLA-4 inhibitors.

BTK acts as a critical downstream effector within the BCR signaling axis. It is indispensable for maintaining B cell survival and normal activation. Transgenic mice with B cell-specific BTK over-expression (CD19-hBtk) develop spontaneous GCs and anti-nuclear antibodies, recapitulating key pathological features of pSS. Functional assays using cells from these transgenic animals show that BTK-overexpressing B cells upregulate IFN-γ and ICOS expression in T cells, and facilitate follicular helper T (Tfh) cell differentiation (63).

Elevated BTK expression is detected in circulating B cells from patients with active pSS. This molecular alteration correlates closely with glandular lymphocytic infiltration (64). In peripheral B cells, BTK expression levels are tightly coupled to its phosphorylation status, and enhanced BTK activity is prevalent among pSS patients. Higher BTK abundance corresponds to increased autoantibody titers and aggravated T cell infiltration in parotid glands. Notably, abatacept can reverse abnormal BTK expression in patient-derived B cells (65).

Abatacept exerts its B cell-modulating functions via two primary pathways: indirect regulation of BCR signaling, and direct targeting of BTK, the core mediator of this signaling cascade (**Figure 1**). Additional investigations are required to define the biological roles of other B cell-associated molecules.

A substantial proportion of pSS patients develop germinal center-like structures (GC-LSs) within glandular tissues. These lesions are distinguished by high expression of activation-induced cytidine deaminase (AID), a key enzyme responsible for immunoglobulin somatic hypermutation (SHM) and class switch recombination (CSR). AID accumulates extensively in follicular dendritic cell (FDC) networks of ectopic GCs, yet it is undetectable in healthy salivary glands (SGs). Within AID-positive lesions, local B cells undergo continuous proliferation, somatic hypermutation and class switching (66).

Studies using BXD2 autoimmune mouse models provide valuable mechanistic insights. Blockade of the CD28-CD86 co-stimulation pathway with AdCTLA-4-Ig restores physiological AID expression and reduces the production of pathogenic IgG autoantibodies (67). Clinical observations in human cohorts further confirm that CTLA-4-Ig effectively inhibits ectopic germinal center formation in pSS patients.

Rituximab, a widely used anti-CD20 monoclonal antibody, yields variable clinical outcomes in pSS management. Its limited therapeutic efficacy is largely attributed to the unique properties of B cells residing within ectopic lymphoid structures (ELS). B cells in ELS sustain autonomous activation, which cannot be fully eliminated by B cell-depleting agents. Failure to eradicate gland-resident B cells and ectopic GCs ultimately leads to treatment refractoriness (68).

AID-driven B cell hyperactivation represents a promising therapeutic target for human autoimmune diseases. CTLA-4-Ig is capable of directly suppressing AID activity (67). Combined anti-CD20 and CTLA-4 targeted therapy may exert synergistic anti-autoimmune effects. This combinatorial strategy holds potential to overcome drug resistance and improve clinical outcomes for pSS patients.

### 2.6 Regulation of T-cell subsets by CTLA-4 in pSS

CTLA-4 is a key functional molecule on T-cells. In particular, Treg cells—an important T-cell subset—are characterized by constitutively high CTLA-4 expression, which is closely linked to Treg homeostasis and function in patients with pSS. Conventional CD4+CD25high regulatory T cells are decreased, whereas CD4+CD25lowGITR+ cells are expanded in the peripheral blood of pSS (69). These CD4+CD25lowGITR+ cells, also displaying Treg phenotype and function, are present in SG inflamed tissues and are expanded in the peripheral blood of subjects with inactive disease (69). The frequency of circulating Treg cells is significantly reduced in patients with relapsing pSS, particularly those with high disease activity (70). Similarly, markedly decreased Treg numbers have also been observed in the salivary glands of pSS patients (71). In a spontaneous pSS mouse model (C57BL/6. non-obese diabetic (NOD)-autoimmune exocrinopathy (Aec)1Aec2), Tregs from salivary gland-draining lymph nodes exhibit elevated CTLA-4 expression, a higher proportion of activated Tregs, and a lower proportion of resting Tregs compared with Balb/c mice (72). In the B6.NOD-Aec mouse model, Tregs directly damage salivary gland epithelial cells and exacerbate SS lesions via upregulated IFN-γ and IL-17 production (72).

Besides constitutive expression in Treg subsets, CTLA-4 can also be detected in other T-cell populations. Tfh cells are a specialized subset of CD4+ T-cells that drive germinal center B cells to produce high-affinity antibodies. By contrast, Tfr cells are a recently identified specialized effector subset of Treg cells that function to suppress excessive B cell responses (73). Tfh cells have attracted considerable attention due to their critical roles in the formation of ELS in patients with pSS, which resemble GCs and support robust autoreactive B cell responses (74), the primary pathological function of Tfh cells in SS is to promote the activation and differentiation of self-reactive B cells. Tfh cells have been detected in the SGs of pSS patients and shown to facilitate B cell maturation (75). Tfh cells mainly mediate T cell-dependent B cell immune responses by secreting interleukin-21 (IL-21). This cytokine is a key molecule that drives B cell activation and differentiation into plasma cells (76).Further studies on pSS have revealed that abatacept can not only markedly regulate circulating follicular helper T (cTfh) cells, but also inhibit B cell activity. This is manifested by decreased serum autoantibody titers, reduced proportions of peripheral plasmablasts, and downregulated expression of BTK in B cells (65, 77).

Tfr cells are thought to play an essential protective role, as deficiency of Tfr cells leads to prominent lymphocyte infiltration and antibody deposition in the SGs of mice with experimental SS (78). Notably, both Tfh and Tfr cells express CTLA-4, with markedly higher expression observed in Tfr cells (79). Approximately 80% of Tfr cells express CTLA-4, a proportion similar to that of ICOS+CXCR5- Treg cells (CD4+ICOS+CXCR5-Forkhead box protein P3 (Foxp3)+) (79). In comparison, only about 20% of Tfh cells and ICOS+ non-Treg cells (CD4+ICOS+CXCR5-Foxp3-) are positive for CTLA-4, whereas CD4+ICOS-CXCR5-Foxp3- (DN) cells, which mainly consist of naive T-cells, show almost no CTLA-4 expression (79). Deletion of CTLA-4 in adult mice resulted in increased numbers of both Tfh and Tfr cells and enhanced humoral immune responses. In the effector phase, loss of CTLA-4 on Tfh cells amplified B cell responses, whereas loss of CTLA-4 on Tfr cells impaired the suppression of antigen-specific antibody production. Studies have also demonstrated that non-Tfr Treg cells can suppress B cell responses through CTLA-4. Moreover, Treg and/or Tfr cells may downregulate B7.2 expression on B cells outside GCs as a suppressive mechanism. Within GCs, however, Tfr cells exert potent suppression of B cells via CTLA-4 through a pathway independent of B7.1 or B7.2 modulation (79). Collectively, CTLA-4 plays multifaceted regulatory roles in controlling the functions of Tfh, Tfr, and Treg cells, thereby orchestrating humoral immune homeostasis (79) (**Figure 1**).

### 2.7 Genetic polymorphisms of CTLA-4 and susceptibility to pSS

Beyond immune cell dysregulation, genetic factors also contribute to abnormal CTLA-4 signaling in pSS. Genetically, a haplotype refers to a set of closely linked genetic polymorphisms on a single chromosome that are stably inherited as a unit. The CD28 GC haplotype represents a specific allelic combination of polymorphic loci within the CD28 gene region; similarly, CTLA-4 CAG and CGA haplotypes are distinct haplotype variants of the CTLA-4 gene. These haplotypes can regulate the transcriptional level and protein function of CD28 and CTLA-4, thereby affecting immune homeostasis and disease susceptibility. From a genetic perspective, a study in a Mexican population by López-Villalobos et al. demonstrated that the CD28 GC haplotype was associated with reduced susceptibility to pSS, whereas the CTLA-4 CAG and CGA haplotypes were identified as significant risk factors (80). Genetic regulation of CD28, CTLA-4, and local immune responses in glandular tissues may influence gene expression in patients with pSS (80). Additionally, The CTLA-4 + 49G/A and CT60 haplotypes are linked not only to disease susceptibility but also to extraglandular manifestations (81). Primary SS is associated with both the +49A;CT60A and +49A;CT60G haplotypes, whereas the +49G;CT60G haplotype is protective against pSS (81). The +49A;CT60G haplotype association is predominantly with Ro/La autoantibody-positive pSS, and the dose of this haplotype influenced the severity of daytime sleepiness (81). The +49A;CT60A haplotype appears to be protective against Raynaud’s phenomenon in pSS patients (81). Nevertheless, studies on CTLA-4 gene polymorphisms have yielded conflicting results. Gottenberg et al. detected no significant association between CTLA-4 CT60 or +49A/G polymorphisms and pSS in two independent Caucasian cohorts with a validation design, and pooled analysis further confirmed the lack of association, suggesting that sampling bias and insufficient replication may account for false-positive outcomes in earlier reports (82). The discrepancies between these studies may be attributed to differences in research strategies: Gottenberg et al. focused on individual polymorphisms and used a replicated case-control design, whereas Downie-Doyle et al. analyzed combined haplotypes and performed phenotypic stratification, which may uncover subgroup associations that are not observed in unstratified, single-locus analyses.

Notably, a recent study by Feng et al. further expanded the understanding of CTLA-4 genetic variations in pSS by investigating novel single nucleotide polymorphisms (SNPs) (83). They demonstrated that the C allele and CC genotype of CTLA-4 rs733618T/C were significantly more prevalent in pSS patients. In addition, the rs733618T/C polymorphism was significantly associated with anti-SSA positivity, anti-Sjögren’s syndrome antigen B (SSB) positivity, and leukopenia, while rs16840252T/C was related to ANA positivity. These results are consistent with the positive associations reported by Downie-Doyle et al, but differ from the negative conclusions of Gottenberg et al. Such inconsistencies may result from the analysis of distinct SNP loci, ethnic differences, sample size disparities, and whether haplotypes or individual loci were evaluated. Collectively, these findings indicate that the association between CTLA-4 polymorphisms and pSS is likely locus-specific rather than universal, and certain CTLA-4 variants may contribute to pSS susceptibility by affecting immune balance, autoantibody production, and clinical phenotypes. These genetic variations may affect CTLA-4 and CD28 transcription and protein expression in salivary gland tissues. At the transcriptional and protein expression levels, analyses of salivary gland tissues from patients with pSS have revealed significantly upregulated CTLA-4 expression (84). In contrast, CD28 expression is markedly decreased in pSS patients (5).

### 2.8 Preclinical evidence for targeting CTLA-4 in SS

Based on the above immunological findings, preclinical studies using mouse models of SS have further validated the therapeutic potential of targeting CTLA-4. Administration of adeno-associated virus type 2 (AAV2)-delivered CTLA-4-IgG fusion protein reduces inflammatory cell infiltration in glandular tissues, decreases inflammatory cytokine levels, and alleviates sicca symptoms in mice (85). Moreover, Adenovirus-mediated CTLA-4-IgG can up-regulate AQP5 expression in mouse submandibular glands and effectively suppress the development of SS in mice (86). Thus, targeting the CTLA-4 checkpoint represents a promising strategy for pSS patients, although further clinical investigation is warranted. Clinical studies on immune regulation via the CTLA-4 checkpoint for the treatment of SS remain limited, with current research primarily focusing on abatacept.

### 2.9 Clinical efficacy of abatacept in pSS

Several studies have primarily investigated the effects and mechanisms of abatacept in pSS patients, and key clinical trial data are summarized in **Table 1**. Abatacept treatment significantly reduces the numbers of lymphocytic foci and local FoxP3+ T-cells of glandular inflammation in pSS patients, and increases saliva production (93). The abundant Tfh cells in the peripheral blood and SGs of pSS patients correlate with extensive lymphocytic infiltration of the SGs and high disease activity scores (94).

**Table 1:** Summary of abatacept treatment for Sjögren’s syndrome.

Researchers	Time	Research type	Patients	Diagnosis	Result and conclusion
Machado AC (87)	2020	Prospective cohort study, no control group	11	Primary Sjögren’s syndrome	Statistically significant improvements were observed in the ESSDAI score, unstimulated salivary flow rate, and the emotional role functioning domain of the SF-36. No improvements were observed in ocular parameters (Schirmer test, tear film break-up time, and ocular staining score) or in the FACIT index. Abatacept therapy improved xerostomia and systemic disease activity.
Meiners PM (88)	2014	Prospective cohort study, no control group 24 weeks of treatment, 48 weeks of observation	15	Early primary Sjögren’s syndrome	ESSDAI, ESSPRI, rheumatoid factor, and IgG levels decreased significantly during treatment, while salivary and lacrimal gland function remained unchanged. Fatigue and HRQoL improved significantly. Abatacept treatment is effective, safe, and well tolerated and results in improving disease activity, laboratory parameters, fatigue, and HRQoL in patients with early and active pSS.
Tsuboi H (89)	2023	Prospective cohort study, no control group 52 weeks	68	Sjögren’s syndrome (combined with rheumatoid arthritis)	SDAI, ESSDAI and ESSPRI decreased. Patients with SDAI remission increased. Saliva volume and Tear volume increased. Abatacept ameliorated both glandular and extraglandular involvements, as well as the systemic disease activities and patient-reported outcomes based on composite measures.
van Nimwegen JF (90)	2020	Single-centre, Randomized, double-blind, placebo-controlled, phase 3 trial 24 weeks	80	Early active primary Sjögren’s syndrome	Primary outcome did not significantly differ. No recommendation of abatacept as standard of care
Baer AN (91)	2021	Randomized, double-blind, placebo-controlled, 169 days	187	Active primary Sjögren’s syndrome	The primary endpoint (ESSDAI), secondary endpoints(ESSPRI, SWSF), other clinical and PRO endpoints showed no statistically significant difference in the abatacept treatment group compared with the placebo group. Abatacept treatment did not result in significant clinical efficacy compared with placebo in patients with moderate-to-severe pSS.
de Wolff L (92)	2022	Randomized, double-blind, placebo-controlled, 48 weeks	40	Primary Sjögren’s syndrome	ESSDAI and ESSPRI scores improved, with 50% of patients achieving low disease activity levels, dry eye and laboratory tests improved, and 73% of patients achieving a CRESS response. Abatacept treatment has shown improvements in clinical, patient-reported, dry eye and laboratory outcomes

ESSPRI, EULAR Sjögren’s Syndrome Patient Reported Index; ESSDAI, EULAR Sjögren’s Syndrome Disease Activity Index; SWSF, Stimulated whole salivary flow; CRESS, Composite of Relevant Endpoint for Sjögren’s Syndrome Response; FACIT, Functional Assessment of Chronic Illness Therapy; HRQoL, Health-related quality of life; TBUT, Tear film breakup time; SDAI, Sjögren’s Syndrome Disease Activity Index.

Abatacept treatment in patients with pSS reduces circulating Tfh cell numbers and the expression of the activation marker ICOS on T cells. Lower numbers of activated circulating Tfh cells contribute to attenuate Tfh cell-dependent B cell hyperactivity and may underlie abatacept’s efficacy (77). In the open-label active Sjögren Abatacept Pilot study, abatacept also suppressed the formation of GCs in parotid glands of pSS patients, which depends on costimulation of activated Tfh cells, thereby inhibiting the local formation of autoreactive memory B cells. Presence of GCs at baseline predicts responsiveness to abatacept in the ESSDAI glandular domain (95).

Overall, open-label uncontrolled studies consistently show that abatacept treatment provides certain benefits, including reduced disease activity, improved glandular function, and better quality of life (87–89). However, high-quality randomized double-blind controlled trials yield inconsistent results (91, 92). Baer et al. reported no significant clinical efficacy in moderate-to-severe pSS, whereas de Wolff et al. demonstrated improvements in clinical indices, patient-reported outcomes, and laboratory parameters (91, 92). These discrepancies may be related to differences in disease duration, baseline disease activity, study endpoints, and follow-up duration across trials.

Baseline differences drive divergent therapeutic outcomes in pSS trials. The Abatacept Sjögren Active Patients (ASAP) serial trial recruited exclusively early active pSS patients (88) with positive salivary gland biopsy and disease duration ≤7 years, featuring higher baseline inflammation, no hydroxychloroquine use, and low glucocorticoid exposure (90). Conversely, other trials primarily enrolled advanced, long-standing pSS patients (91). For patients with long disease duration, irreversible glandular damage and even fibrosis may occur.

The insufficiency in trial design and assessment tool further widen result’s disparities. Uncontrolled open-label studies impair result reliability. Short follow-up periods show improvements in reversible serum markers and glandular inflammatory infiltration, failing to reflect long-term outcomes like salivary gland function. Salivary flow measurements lack stability. Subjective ratings in ESSDAI, ESSPRI assessments are susceptible to external factors, and variable salivary flow makes it hard to identify mild therapeutic effects of targeted immunotherapies. The ASAP serial trials deserve close attention. Its early open-label trial suggested some clinical benefits with abatacept, but the study lacked a placebo arm, had a small sample size and insufficient statistical power. Results were therefore easily skewed by the placebo effect and subjective assessment bias.

The main randomized controlled ASAP-III trial adopted ESSDAI as the only outcome measure and found no meaningful clinical response to abatacept, so the drug was not recommended for routine use in general patients (90). However, its 48-week extension trial yielded clear therapeutic effects. This indicates abatacept works gradually: clinical benefits cannot be fully captured within the 24-week short-term follow-up, and sustained long-term use helps relieve symptoms over time (92).

Pathological data from ASAP-II and ASAP-III participants showed that abatacept specifically suppresses germinal center formation in salivary glands. Still, it has limited impact on widespread lymphocytic infiltration and glandular structural damage. Pathological changes occur slowly and do not align well with improvements in clinical symptoms (95).

Related methodological analyses based on ASAP-III further proved that ESSDAI is not sensitive enough and likely underestimates treatment effects. By contrast, the multidimensional composite endpoint CRESS can more accurately reflect the actual clinical value of abatacept (96).

In summary, the inconsistent experimental conclusions stem from multiple factors, including differences in patients’ baseline characteristics (such as disease duration and activity level), sample size, study design, observation period, assessment tools and endpoint indicators. These factors may all affect the research outcomes. For future studies, it is necessary to optimize the design and unify evaluation criteria to obtain more credible results.

## 3 Discussion

The central role of CTLA-4 in the pathogenesis of pSS is well-supported yet incompletely defined. So the therapy efficacy based on the checkpoint is uncertain. Although CTLA-4 targeted therapy for pSS is still in an early stage, and future investigations should focus on precision medicine approaches. Combined with the above content and relevant references, these issues are highlighted and merit further in-depth exploration.

1. Mechanistically, T cell subsets including Trg,Tfh and Tfr are modulated in both animal models and human subjects. Local B cell infiltration as well as the levels of IgG, IgA and rheumatoid factor (RF) are altered to varying degrees. The involvement of T and B cells in the pathogenesis of Sjögren’s syndrome has been validated in both animals and humans. However, the regulatory mechanisms of CTLA-4 on T cell subsets, as well as its effects on upstream and downstream targets of B cell-related signaling pathways such as BCR and BTK, remain to be further investigated.
2. Predicting the efficacy and adverse effects of CTLA-4 blockade or potentiation requires continuous monitoring and assessment. However, no standardized stratification criteria based on predictive biomarkers are available. Patients’ baseline characteristics, along with CTLA-4 polymorphisms, anti-Ro/SSA status, Tfh/Tfr ratios and baseline germinal center presence, are correlated with treatment response and irAEs. Furthermore, the cellular subsets and biomarkers mentioned in previous preclinical and clinical studies, as well as immune-related adverse events themselves and their associated biomarkers such as CD21lo B cells, can also be adopted as indicators for treatment response and risk prediction.
3. Existing studies confirm the involvement of inflammatory pathways in irAEs. Blockade of GM-CSF, IL-6, TNF or IL-23 signal pathways can effectively reduce or prevent ICI-related irAEs. This strategy, in addition to conventional steroid treatment, is worthy of further research.
4. No definitive conclusion has been reached on the efficacy of CTLA-4-Ig for SS. High-quality clinical evidence remains limited. Discrepant results arise largely from inconsistent baseline conditions, disease activity, cohort size, assessment tools, ndpoint definition and observation durations. Differences in immune backgrounds and autoantibody-based subtypes can also cause variable therapeutic effects. Future trials require standardized protocols and refined patient stratification.
5. Studies have demonstrated that CTLA-4 is not an isolated target. Instead, it interacts with other co-inhibitory receptors including PD-1, TIGIT, TIM-3 and LAG-3 to form a multi-layered immune regulatory network. Monotherapies targeting CTLA-4 or PD-1 blockade have achieved unprecedented efficacy in various malignancies. Nevertheless, a large number of patients fail to respond to such treatments, and certain tumor types exhibit intrinsic resistance. Meanwhile, combination regimens have yielded additional therapeutic benefits in some patients. As discussed above, apart from CTLA-4, PD-1 and other co-inhibitory receptors are also expressed in patients with SS. For patients who show unsatisfactory therapeutic effects after single enhancement of CTLA-4 signaling, combined activation of CTLA-4 and other co-inhibitory receptors can be considered to strengthen immune suppression, thereby developing multi-target therapeutic strategies. This approach represents a reverse activation application of combined treatment strategies targeting co-inhibitory receptors.
